# Behavioral techniques for controlling visual stimuli to modulate visual processing in the brain and their implementation in augmented reality technology

**DOI:** 10.3389/fnins.2026.1925212

**Published:** 2026-09-22

**Authors:** Tadamasa Sawada, Maria Dvoeglazova, Alla Zargaryan, Anna Tamrazyan, Valeria G. Kozhevnikova, Asya Berberyan, Matteo Feurra, Elena S. Gorbunova

**Affiliations:** 1 Zaven P. and Sonia Akian College of Science and Engineering, American University of Armenia, Yerevan, Armenia; 2 Department of Psychology, Russian-Armenian (Slavonic) University, Yerevan, Armenia; 3 School of Psychology, HSE University, Moscow, Russia; 4 Faculty of Education Psychology and Sociology, Armenian State Pedagogical University, Yerevan, Armenia; 5 Centre for Cognition and Decision Making, Institute for Cognitive Neuroscience, HSE University, Moscow, Russia

**Keywords:** Augmented Reality (AR), brain stimulation, extended reality, field study, virtual reality (VR), XR

## Abstract

This study reviews the behavioral techniques that control visual stimuli to systematically modulate visual processing in an observer’s brain. These techniques are indirect but more affordable than more-direct techniques for stimulating the brain. The limitations of these techniques and the calibrations necessary for them are also discussed. Some of the behavioral techniques can be also used in field studies by making use of Augmented Reality (AR) technology and a head-mounted display (HMD). The AR HMDs for the behavioral techniques was assembled by combining a smartphone and a wearable stereoscope, both of which were also affordable, as a proof-of-concept. The reviewed behavioral techniques make it possible that researchers with limited budgets can conduct neuroscience studies and that students can learn about these subjects in low-income countries or in underfunded institutions. It can contribute to the democratization of neuroscience and the global equity of scientific research, as well as to broadening the populations studied in cognitive and neuroscience.

## 1 Introduction

There are two major approaches in Neuroscience to test the relationship between the brain activity and the human behavior. One is testing the correlation between the brain activity and the behavior. Several neurophysiological devices have been developed for estimating the brain’s activity on the basis of physical signals that are associated with the brain’s activity, such as the fMRI, EEG, MRI, NIRS, and single cell recording. The other approach is to modulate the brain’s activity while observing changes of the behavior that was caused by the brain’s activity. An advantage inherent in this latter approach is that a causal relationship between the brain activity and the behavior can be tested. Several devices that can directly stimulate the brain and systematically modulate the brain’s activity have been developed. These devices can be used to observe an effect on the behavior caused by the brain’s activity that is induced by the devices.

Non-invasive brain stimulation has advanced rapidly in the past two decades and now offers practical tools to modulate cortical excitability and oscillations in humans. Transcranial magnetic stimulation (TMS) can transiently perturb or bias activity in targeted cortical regions, with offline protocols producing effects that outlast stimulation and can be probed during subsequent tasks; transcranial electrical stimulation (tES, including tDCS and tACS) can shift membrane polarization and entrain ongoing rhythms in a frequency-specific manner (Pozdniakov et al., 2021; Feurra et al., 2019; Dayan et al., 2013; Polanía et al., 2018; Thut et al., 2017; Huang et al., 2005). Note that tES devices are lightweight and have been safely used in portable and home-based, remotely supervised settings, which enables coupling stimulation with naturalistic behavior in the field (Fertonani and Miniussi, 2017; Charvet et al., 2015; Woods et al., 2016). By contrast, TMS remains less amenable to fully mobile deployment because of coil positioning, acoustic output, and safety constraints, but it can still be applied in short offline blocks immediately before field tasks. Together, these approaches provide a causal complement to correlational measures and create a bridge between tightly controlled laboratory studies and ecologically valid testing outside the laboratory.

Beyond non-invasive methods, invasive brain–computer interfaces provide strong causal leverage in humans by directly recording and stimulating cortex, enabling volitional control and communication in clinical populations, though their use is restricted to surgical settings (Hochberg et al., 2012; Willett et al., 2021). In animals, optogenetics offers unmatched cell-type and timing specificity for activating or silencing defined circuits, setting mechanistic benchmarks for human neuromodulation studies (Deisseroth, 2011). Complementarily, classical lesion studies and modern lesion-network mapping in patients link focal damage to distributed network dysfunction, supplying convergent causal evidence at the systems level that can guide hypotheses tested with non-invasive stimulation such as in AR-enabled, naturalistic contexts (Fox, 2018).

There are also behavioral techniques that use properties of an image to modulate visual processing in certain brain regions when the image is shown to an observer. For example, the visual information of an isoluminant image is mainly processed in the ventral pathway while the dorsal pathway is mostly blind to the isoluminant information (see the subsection “2.1 Isoluminant stimuli and the dorsal pathway”). This allows us to test the role of these pathways in behavioral tasks based on visual information. Note that the isoluminant image can theoretically represent the same amount of visual information as the gray-scale image. So, visual processing in the dorsal pathway can be preferentially suppressed. Such a behavioral technique is indirect but some of the regions whose activity can be modulated by the behavioral techniques are difficult to be selectively stimulated by the current physical techniques because of the positions of the regions (e.g., superior colliculus, see the subsection “2.3 S-cone isolating stimuli and the superior colliculus”). They can be also incorporated with the physical techniques to test interactions between multiple regions of the brain (Kveraga et al., 2007; Thomas et al., 2012). Importantly, the behavioral techniques are more affordable compared with most of neurophysiological devices. Researchers with limited budgets can conduct neuroscience studies and that students can learn about these subjects in low-income countries or in underfunded institutions. It can contribute to the democratization of neuroscience and the global equity of scientific research (see Jurcik et al., 2024; Papoutsaki et al., 2016; Valliappan et al., 2020; Bhamla et al., 2017; Wei et al., 2013; Cybulski et al., 2014; Rohof et al., 2020 for studies of research tools with similar motivations). It can also contribute to broadening the populations studied in cognitive and neuroscience (Henrich et al., 2010; Nielsen et al., 2017; White and Muthukrishna, 2026).

Some of the behavioral techniques can be used in field studies by making use of Augmented Reality (AR) technology and a head-mounted display (HMD) if they can be implemented as computer image filters that can be applied to any images. A camera captures an image of a real environment, the filter of the behavioral technique is applied to the captured image, and the filtered image is shown to an observer who is wearing the HMD in real-time or near real-time just as if the observer is seeing the scene directly [see also Kleinholdermann et al., 2009 for a study using one of the behavioral techniques modulating the brain activity in a virtual reality (VR) set-up]. Note that the AR technology has been used to study the cognitive system in the field outside the laboratory with image filters that modify the visual information of the scene (e.g., Anstis, 1992; Bao and Engel, 2019; Grush et al., 2015; Juan and Calatrava, 2011; Krösl et al., 2020; Velázquez et al., 2015; Tsujita et al., 2023; Sawada et al., 2022; Farshchi et al., 2021). Sensory information from a real environment is noisy, complex, and dynamic in field studies, and such conditions are usually difficult to reproduce in laboratory settings. Field studies can therefore complement knowledge from laboratory experiments by providing greater ecological validity, whereas laboratory studies have the advantage of greater experimental control. It helps us to assess whether or how much findings from the laboratory studies can be generalized in a real environment (see Hengartner, 2018). Thus, the combination of the behavioral techniques and the AR technology can bridge the gap between tightly controlled laboratory studies in neuroscience and ecologically valid testing in the field (see Boto et al., 2018; Maeda et al., 2005; Aoyama et al., 2015 for other technical approaches aimed at bridging this gap in neuroscience).

This study will review the behavioral techniques that can systematically modulate visual processing in certain regions of the brain. Note that these techniques have not been summarized in any organized manner and there are no common keywords to find them. This study will provide a comprehensive list of the techniques. Some of the techniques were also implemented as image filters and they were installed on AR HMDs as a proof-of-concept.

## 2 Behavioral techniques to systematically modulate visual processing in the brain

This study is a narrative review intended to survey behavioral techniques to systematically modulate visual processing in certain regions of the brain (Table 1). These techniques have been used in cognitive science and neuroscience. All of the techniques in the review were originally identified based on the authors’ knowledge of these fields. A summary of these techniques was provided to the AI Large Language Model (LLM, Chat-GPT5.6 Sol) to ascertain whether there were any other techniques, but no particularly valid ones were suggested by the LLM.

**TABLE 1:** A list of behavioral techniques that can systematically modulate visual processing in the brain.

Behavioral techniques to systematically modulate visual processing in the brain			
Method	Brain region	Calibration	Additional hardware
Isoluminance*	Dorsal pathway	Cumbersome	–
Spatial frequencies	Magnocellular and Parvocellular pathways	Relatively easy	Good processor for Fourier and inverse-Fourier transforms
S-cone isolation	Superior colliculus	Costly and cumbersome	–
First-order and Second-order noise*	Primary visual cortex (V1)	Relatively easy	–
Power-law	Primary visual cortex (V1)	Relatively easy	–
Regions in the retinae	Left and right cerebral hemispheres	Depending on eye tracker	Eye tracker

*These techniques were implemented as image filters for AR HMDs in this study (see the section “3 AR implementation of some of the behavioral techniques”).

### 2.1 Isoluminant stimuli and the dorsal pathway

The ventral and dorsal pathways of the cortex are often referred to as the “what” and “where” pathways. The ventral “what” pathway processes the colors, shapes, and sizes of individual objects while the dorsal “where” pathway processes the positions and motions, as well as the interaction of an observer with the objects (see Goodale and Milner, 1992; Mishkin et al., 1983; Kravitz et al., 2011; McIntosh and Schenk, 2009 for reviews and discussions). Note that there are some interactions between these pathways (e.g., Schenk, 2012; de Haan and Cowey, 2011; Cardoso-Leite and Gorea, 2010; Schenk and McIntosh, 2010; Doniger et al., 2002; Milner, 2017; Borst et al., 2011; Ray et al., 2020) but the functions of the pathways are well-separated from one another. This difference between the functions of the pathways can be used to selectively study their roles in visual perception.

A retinal image can be decomposed into two 2D distributions of achromatic luminance and isoluminant- (equiluminant-) chromatic information. The luminance information is processed in both ventral and dorsal pathways while the isoluminant chromatic information is processed primarily in the ventral pathway. If the retinal image itself is isoluminant, the image can only stimulate the ventral pathway while the dorsal pathway is only minimally stimulated by this image. This technique using the isoluminant image has been used in many psychophysical studies to examine the roles of the ventral and dorsal pathways and their results are usually consistent with the results of neurophysiological studies that test the ventral and dorsal pathways.

This selectivity of the dorsal pathway is attributed to its input. The input of the dorsal pathway is mainly from the magnocellular pathway, which processes the achromatic luminance information in the retinal image, but also from the parvocellular and koniocellular pathways, which process the chromatic information in the retinal image (Goodale and Milner, 1992). The ventral pathway also receives its input from these three cellular pathways while its main source of input is the parvocellular pathway. Also, the magnocellular cells are not completely blind to isoluminant chromatic change (Schiller and Colby, 1983; Logothetis et al., 1990). So, the selective stimulation of the ventral pathway by using isoluminant image is a matter of degree (see Goodale and Milner, 1992; Skottun, 2013).

An isoluminant image is often generated with the red-green isoluminant scale that is based on the red and green channels of an RGB display (Figure 1). This red-green isoluminant scale is represented as a 1D sub-space in a 2D isoluminant color space, which is also a sub-space in a 3D color space. This one-dimensionality makes it easier to control the RGB values of the isoluminant image. Note that the grayscale values of the grayscale image are also represented in a 1D space. This consistency of dimensionality is convenient when the perception of the red-green isoluminant image is compared with the perception of a grayscale image as a control. On the other hand, the number of levels in the red-green scale tends to be smaller than in the grayscale. This discrepancy can be addressed by decreasing the level number in the grayscale. The control image can be generated by removing the red or green channels of the isoluminant image so that the image is represented by the red-black or the green-black scales. The red-black and the green-black scale are also represented as 1D sub-spaces in the 3D color space and they are correlated with the 1D sub-space of the grayscale. The number of isoluminant levels can be larger by using the red-green scale than by using the blue-red or the blue-green scales because the range of luminance within each of the red and green channels is larger than the range of luminance within the blue channel.

**FIGURE 1:**
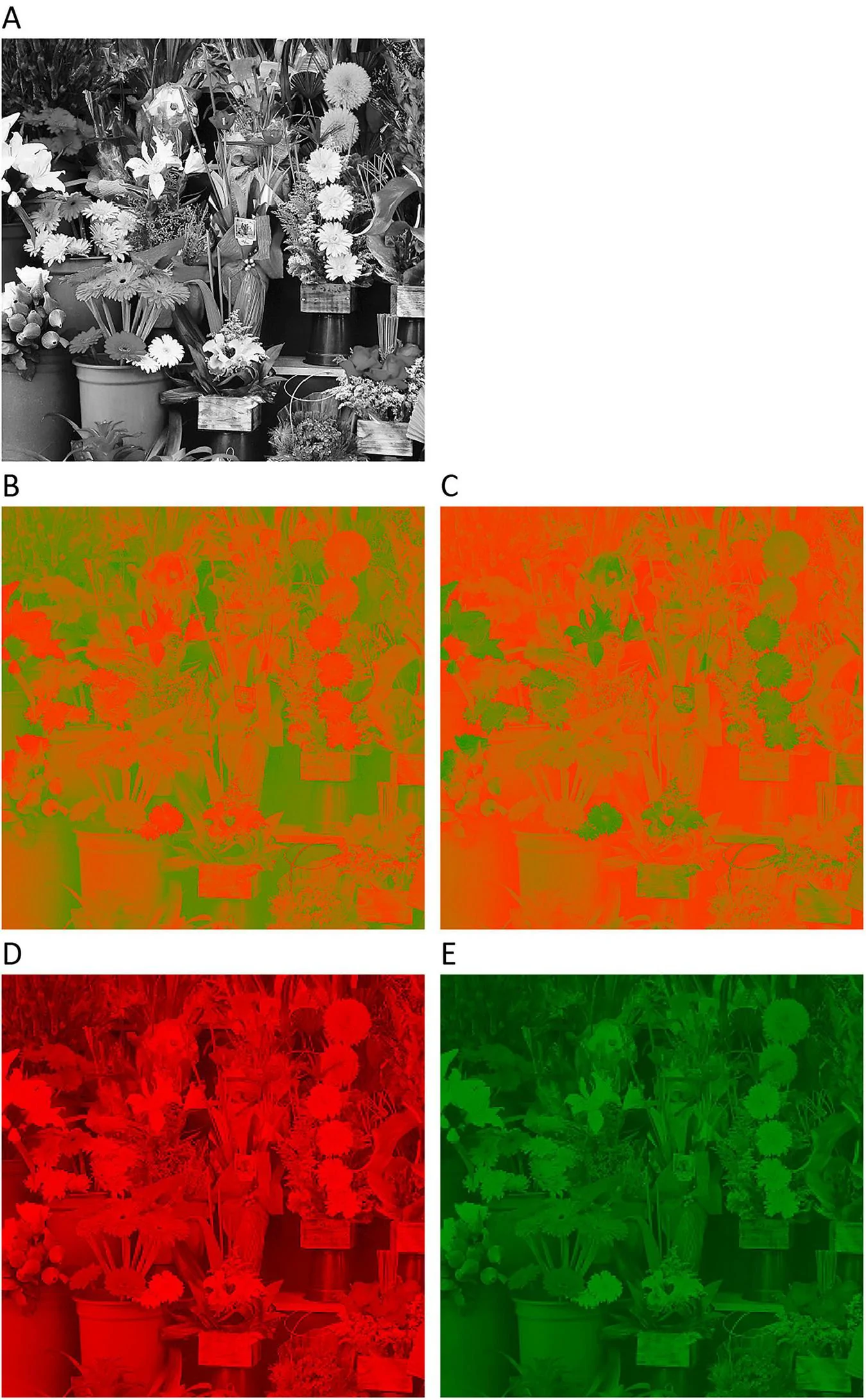
**(A)** A grayscale image of a real scene and **(B,C)** its red-green isoluminant images. **(D,E)**. Red-black scale and green-black scale images that were generated by removing the green channel from the isoluminant image **(B)** and the red channel from the isoluminant image **(C)**. The relationships between the red-green scale and the grayscale in the two isoluminant images **(B,C)** are opposite from one another.

It is necessary to calibrate the red and green channels of a display if you want to generate a red-green isoluminant image. The luminance of each pixel is calculated as the sum of the luminance of the red and green channels that is represented as a monotonically-increasing function of the value of the channel. The function is often characterized by a gamma curve (see also Xiao et al., 2011; To et al., 2013 for discussions about non-gamma curves for LCD displays). The display can be calibrated by estimating (i) the gamma curves of the channels and (ii) the relative scale between the channels. Once this is done, the red and green values of the pixel are controlled so that the sum of the luminance of the channels is constant, or approximately constant, across the image. Once the display is calibrated, the technique can be implemented as an image filter. The image filter was included in an AR app that was developed in this study.

The luminance is a psychophysical variable and the isoluminant scale is different across observers. Then, the display is ideally calibrated for the individual observers and behavioral measurements can be used for the individual calibration. Several simple behavioral methods have been proposed for the display calibration when a 1D isoluminance scale is used to generate the isoluminant image (Anstis and Cavanagh, 1983; Chaudhuri and Albright, 1990; Cavanagh et al., 1984; Liu et al., 2024; To et al., 2013; Wagner and Boynton, 1972; see Murray et al., 2022; Zaman et al., 2023 for the calibration of HMDs). A potential limitation of the behavioral calibration is that the behavioral tasks during the calibration can be challenging for naïve observers who do not have any experience of psychophysical experiments.

If it is difficult to calibrate a display for individual observers, a common setting of the display calibration can be used across the observers. This common setting can be based on physical luminance measurements obtained with a luminance photometer.^1^ If such measurements are not available, a behavioral calibration can be conducted by multiple experienced observers and a single calibration setting is derived from their results. Note that an isoluminant image under the common calibration is not exactly isoluminant for each participant and there can be residual luminance cues. This artifactual effect of the individual difference can be reduced by adding spatial-temporal luminance noise to mask the luminance cues (Birch et al., 1992; Baker et al., 1998; Mollon and Reffin, 1989; Regan et al., 1994).

Note that the calibration is a matter of degree. The effect of the behavioral technique on human behavior should be discussed by comparing it with behavior in a control condition. For the isoluminant image technique, it can be a grayscale image from which the isoluminant image is generated or monochrome-red or -green images (Figures 1D, E) that are generated by extracting either of the red or green channels from the isoluminant image. This comparison also allows us to rule out any artifactual effects caused by the AR system. The artifactual effects caused by the AR system should be considered for all the behavioral techniques that are implemented as image filters of the AR system (Table 1).

### 2.2 Spatial frequencies and the magnocellular and parvocellular pathways

Neurons of the magnocellular pathway in the lateral geniculate nucleus (LGN) are more sensitive to the low spatial and high temporal frequencies of a retinal image, whereas neurons of the parvocellular pathway in LGN are more sensitive to the high spatial and low temporal frequencies (Derrington and Lennie, 1984; Livingstone and Hubel, 1987, 1988; Denison et al., 2014; Wen et al., 2021; see Skottun, 2015 for a review). This difference in spatial frequency tuning between the pathways may be used to stimulate one pathway more than the other pathway (see Skottun, 2015 for a discussion).

The low and high spatial frequency components can be extracted from a grayscale image of a real scene from a camera by applying low-pass and high-pass filters in the 2D Fourier domain of the image (Figure 2). To apply these filters, the 2D Fourier transform of the image is first computed. To generate the low-pass filter, the magnitudes of high spatial frequency components are set to zero in the Fourier domain and the inverse-Fourier transform is computed. To generate the high-pass filtered image, the magnitudes of low spatial frequency components are set to zero in the Fourier domain and the inverse-Fourier transform is computed. The transformed image should be shown on a calibrated display.

**FIGURE 2:**
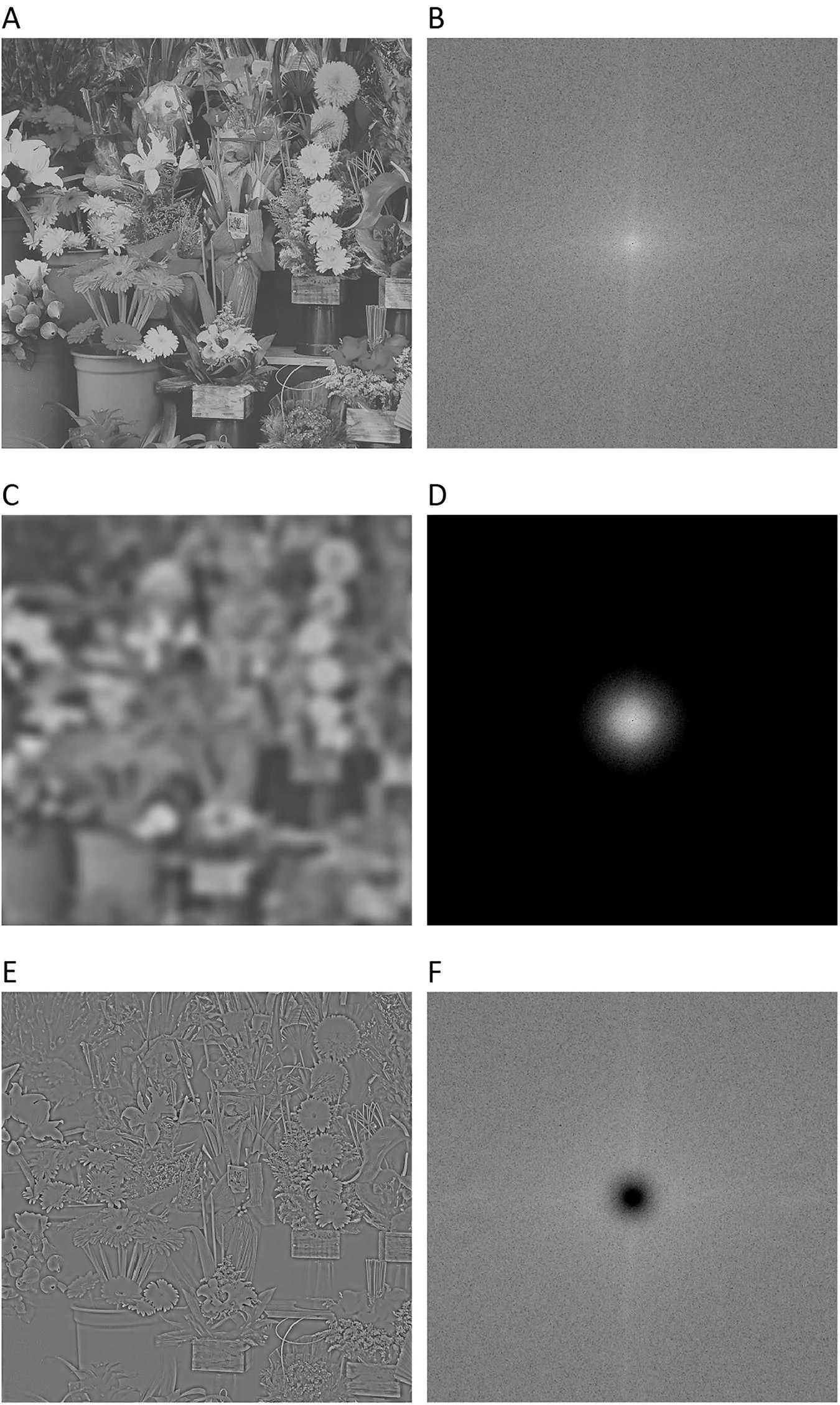
**(A)** A grayscale image of a real scene, its **(C)** low-pass and **(E)** high-pass filtered images, and **(B,D,F)** their 2D Fourier transforms (amplitude spectrums). The low-pass and high-pass filters are 2D Butterworth filters (Butterworth, 1930).

Note that it is not clear how much the processing of a retinal image in the pathways can be suppressed by the low-pass and high-pass image filters alone because the difference in spatial frequency turning between the magnocellular and parvocellular pathway is small (Skottun, 2015). The distinction between the pathways may be enhanced by introducing dynamic changes to the image and controlling its temporal frequency (Gao et al., 2025; Zhou et al., 2025). The activity of the magnocellular pathway can be suppressed by replacing the grayscale of the image with the isoluminant chromatic scale (see the subsection “2.1 Isoluminant stimuli and the dorsal pathway”).

The calibration of the grayscale level of a display is necessary for this behavioral technique (see the subsection “2.1 Isoluminant stimuli and the dorsal pathway”). The relationship between the luminance and the grayscale level is represented as a monotonically-increasing function. The function is estimated based on physical luminance measurements obtained with a luminance photometer or based on a behavioral method by characterizing the function using a gamma curve (To et al., 2013).

These low-pass and high-pass image filters can be used in an AR app (e.g., Gao et al., 2025; Zhou et al., 2025) but the image filters are ideally applied in the Fourier domains of images. It requires the Fourier and inverse-Fourier transforms, which are computationally expensive. A good processor is necessary to apply the filters to an image of a real scene from a camera in real time or near-real time for their AR implementation. The low-pass filter can be replaced with a 2D Gaussian filter, whose mathematical properties are analogous to the properties of the low-pass filter. The high-pass filter emphasizes edges in the images but the high-pass filter may or may not be fully replaced with edge detection algorithms (e.g., Sawada et al., 2022; Farshchi et al., 2021) because the edge detection algorithms often include linear filters whose mathematical properties are different from the properties of the high-pass filter (Marr and Hildreth, 1980) and also because the algorithms often include non-linear processes. It is ideal to also include a grayscale image in the AR system as a control. There can be artifactual effects caused by the AR system and they can be ruled out by comparing the control condition of the grayscale image with the test conditions of the low-pass and high-pass images.

### 2.3 S-cone isolating stimuli and the superior colliculus

The superior colliculus is a part of the mammalian midbrain and its activity can be suppressed by controlling the colors of visual stimuli. The superior colliculus is involved in visual detection, spatial attention, and eye movements (Krauzlis et al., 2013; Kato et al., 2011; Bell et al., 2006; Dorris et al., 1997; Kustov and Lee Robinson, 1996; Spering and Carrasco, 2015; Mizzi and Michael, 2014; Anderson and Carpenter, 2008; Sumner et al., 2002), as well as body movements (Gandhi and Katnani, 2011; Prabhu and Himmelbach, 2023). The superior colliculus is especially sensitive to negatively-affective visual information (Almeida et al., 2015; Wang et al., 2020; Ajina et al., 2020; see also Carretié et al., 2021; Méndez et al., 2022). It is also suggested that the superior colliculus plays a role in integration of visual information from the left and right halves of the visual field (Savazzi and Marzi, 2004; Leh et al., 2010; van Koningsbruggen et al., 2017) and in integration of multisensory spatial information (Leo et al., 2008; Maravita et al., 2008). These results suggest that the superior colliculus is important for preconscious rapid detection and reaction to potential threats.

The superior colliculus receives visual information from the cortex and also from the retinotectal pathway that bypasses the lateral geniculate nucleus (LGN) and the cortex (see May, 2006; Liu et al., 2022 for reviews). Neurophysiological studies in monkeys suggest that the information from the cortex to the superior colliculus depends primarily upon the magnocellular pathway (Schiller et al., 1979). Neither the magnocellular nor retinotectal pathways process chromatic information (Schiller and Malpeli, 1977; Schiller et al., 1979; Marrocco and Li, 1977) and they receive no input from short-wavelength sensitive cones (S-cones; de Monasterio, 1978; Tailby et al., 2012). Both the retinotectal and magnocellular pathways receive input from medium- and long-wavelength sensitive cones (M-cones, L-cones) but they cannot process the chromatic information because these pathways do not process the input from the M-cones and the L-cones in an opponent manner. Note that the retinotectal and magnocellular pathways are based on different neurophysiological mechanisms in the retina and these pathways can have different spectrum sensitivity functions.

It is possible to design visual stimuli based on these properties of the superior colliculus so that the superior colliculus is minimally stimulated. The chromatic information contained in the stimulus is represented in the temporal variation and spatial distribution of S-cone responses while M-cone and L-cone responses are kept constant (Hall and Colby, 2013; Leh et al., 2010; Smithson, 2014). The S-cone isolating stimulus is processed in the LGN and in the visual cortex via the koniocellular pathway (Hendry and Reid, 2000; Xu et al., 2001) but the superior colliculus is minimally stimulated by this stimulus. The S-cone isolating stimulus is also isoluminant because the luminance is based on the M-cone and L-cone responses and because their responses are kept constant. Note that the chromatic information can also be represented by the balance between the M-cone and L-cone responses while keeping the isoluminance of the stimulus (see the subsection “2.1 Isoluminant stimuli and the dorsal pathway”). But, keeping the M-cone and L-cone responses is ideal to minimize the stimulation of the superior colliculus because the spectrum sensitivity functions of the retinotectal and magnocellular pathways can be different from one another. So, the M-cone and L-cone responses can change so that one of these two pathways cannot detect the change (like a metamer) but the other pathway can still detect it. The S-cone isolating stimulus, which only represents chromatic information by the S-cone responses should be used to minimally stimulate the superior colliculus.

Note well that the superior colliculus is not totally blind to the chromatic information in a visual stimulus. It has been shown that neurons in the superior colliculus respond to the chromatic information but with a delay when compared to the response to the achromatic information (White et al., 2009; Zhang et al., 2025; see also Ottes et al., 1987; Herman and Krauzlis, 2017). Hall and Colby (2014, 2016) also showed that neurons in the macaque superior colliculus can respond to input from the S-cones as fast as they can respond to the achromatic information if the S-cones are sufficiently stimulated (see also Coubard, 2017 for a commentary on this study). These results are also consistent with human neuroimaging studies. The human superior colliculus is not blind to the chromatic information in a stimulus (Chang et al., 2016) but its activity can still be reduced if there is no achromatic information in the stimulus (Leh et al., 2010). So, the S-cone isolating stimulus can reduce or delay stimulation of the superior colliculus, but it is a matter of degree. To further minimize any effect of the S-cone input, luminance noise can be added to the stimulus to mask the input (Leo et al., 2008; Sumner et al., 2002).

It is possible to make an image filter for an AR app that can generate an S-cone isolating stimulus from a grayscale image of a real scene out there. The filter replaces the luminance gradient of the image with the S-cone response gradient that subtends between blue and yellow. With this image filter, it is possible to test human behavior while minimizing the visual input to the superior colliculus. Note that S-cone isolating stimuli that were used in the past studies to minimize the stimulation of the superior colliculus were very simple. It can be interesting to test how the superior colliculus responds to S-cone isolating stimuli with complex patterns.

A limitation of this technique is the calibration of a display. A spectroradiometer is necessary to calibrate the RGB channels of the display for S-cone isolation. Stimulation of the S-cone should be controlled while the stimulation of the M-cone and L-cone should be kept individually constant for the S-cone isolation. Note that the spectrum energy distributions of the RGB channels are usually so wide that they individually stimulate multiple types of cones. For example, the S-cone is mostly stimulated by the blue channel but the blue channel usually also stimulates the M-cone and the L-cone to some extent. Each channel stimulates the three types of cones while maintaining the proportion of their stimulation and the stimulation of each type of cone is a sum of the stimulations from the RGB channels. Then, the RGB channels can be represented as three vectors in the LMS color space. This allows us to derive how the intensity of the RGB channels should be controlled to change the S-cone stimulation while the M-cone and L-cone are constantly stimulated. If individual differences in the early visual system are considered, there is a behavioral method to measure these differences and transform the LMS space according to the measured differences (Hall and Colby, 2013, 2014). Artifactual effects caused by the individual difference can be reduced by adding spatial-temporal luminance noise to a calibrated image (Birch et al., 1992; Leo et al., 2008; Sumner et al., 2002; Bertini et al., 2008). From the derived intensity of the RGB channels, the RGB values (usually eight bit for each channel) can be computed. The relationship between the intensity of the RGB channels and the RGB values is not usually linear.

A spectroradiometer is often expensive and it is a limitation of the S-cone isolation technique. A remedy for this limitation can be a collaborative network if the S-cone isolation technique is implemented as an image filter of an AR HMD. The AR HMD is portable and a part of the calibration requiring the spectroradiometer can be conducted remotely by a collaborator who owns the spectroradiometer.

The range of luminance within the blue channel is smaller than the range of luminance within each of the red and green channels. This property of the blue channel limits the number of levels in the scale of the S-cone isolation stimulus. This problem could be addressed by a physical color filter that reduces the intensity of the red and green channels, but not the blue channel as much. The filter would be installed between the screen of an HMD and an observer’s eyes to balance the intensity of the channels.

Note that some past behavioral studies used an isoluminant stimulus with a different hue as a control of the S-cone isolation stimulus (Anderson and Carpenter, 2008; Leo et al., 2008; Bertini et al., 2008). The same idea can be used to generate an image filter that serve as a control of the S-cone isolation filter (e.g., a red-green isoluminance filter).

### 2.4 First-order and second-order noise stimuli and the primary visual cortex (V1)

Many neurons in the primary visual cortex (V1) are sensitive to a change in luminance amplitude and they systematically respond to spatial or temporal patterns of the luminance amplitude (see Carandini et al., 2005; Hubel and Wiesel, 1977; Sawada and Petrov, 2017 for reviews). This type of visual information is referred to as the first-order information while the second-order information is represented by spatial or temporal patterns of a larger-scale image feature that is represented by a change in luminance amplitude, such as luminance contrast, orientation, scale, and motion (Figure 3). There are several different types of the second-order visual information but the most common type is that a spatial pattern is represented by a change of contrast of a random noise pattern or of a regular pattern. It has been considered that the neurons in V1 could not detect the second-order information and that the second-order information could be used to control spatial or temporal patterns without systematically stimulating the V1 neurons. But, neurophysiological studies in monkeys showed that some of the V1 neurons are also sensitive to the second-order information while the proportion of cells sensitive to the second-order information depends on the studies (Lamme et al., 1993; Marcar et al., 2000; O’Keefe and Movshon, 1998; von der Heydt and Peterhans, 1989; Zhou and Baker, 1993; Baker and Mareschal, 2001; Ju et al., 2022; An et al., 2014). This diversity can be partially attributed to the types of second-order information, but it is still observed across studies using the same type of second-order information (luminance contrast, Zhou and Baker, 1993; Baker and Mareschal, 2001; Ju et al., 2022; O’Keefe and Movshon, 1998; An et al., 2014). The neurons are tuned to the spatial properties of second-order patterns that are close to the spatial properties of first-order patterns to which they are also tuned but this does not mean that V1 and the visual system equally processes the first-order and second-order information.

**FIGURE 3:**
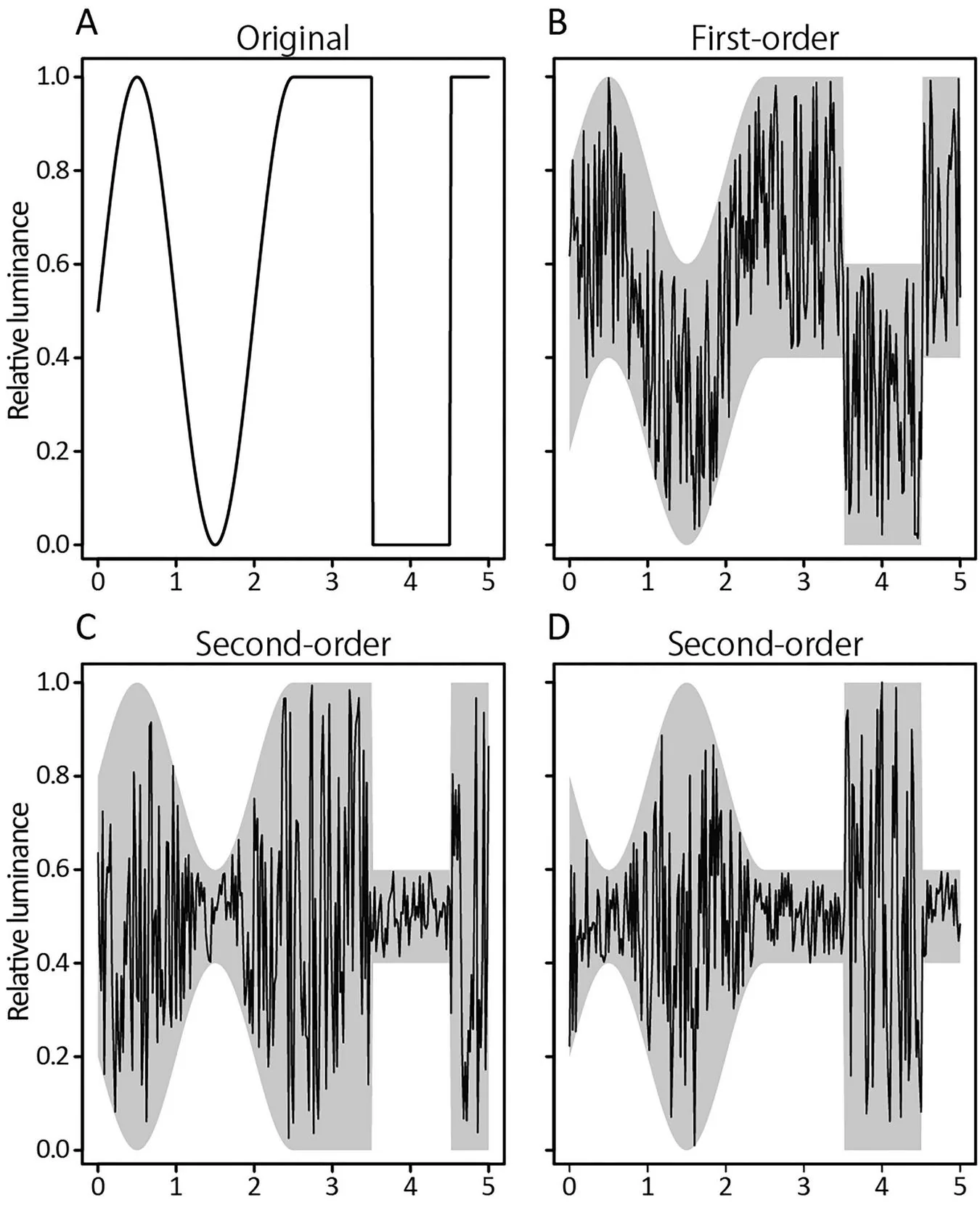
**(A)** A 1D pattern of signal and **(B)** first-order and **(C,D)** second-order noise patterns that represent the signal **(A)**. The distribution of the noise is uniform (white noise) within a range of gray areas in panels **(B–D)**. **(B)** The center of the noise range is proportional to the signal. **(C)** The width of the noise range is proportional to the signal. **(D)** The width of the noise range is negatively proportional to the signal.

V1 neurons are often modeled as a combination of (i) a process that characterizes 2D patterns to which the neurons are tuned to and (ii) a divisive normalization that represents suppression (or facilitation) from LGN, from intracortical connections within V1, and from top-down feedback (see Carandini et al., 2005; Carandini and Heeger, 2012; Heeger, 1992; Sawada and Petrov, 2017 for reviews). The first process is formulated as a 2D linear filter to a retinal image that is followed by a non-linear transformation. This process characterizes sensitivity of the V1 neurons to the first-order visual information while the process cannot detect the second-order information. Physiological studies suggest that the sensitivity of the V1 neurons to the second-order visual information can be attributed to the process that is formulated as the divisive normalization (Tanaka and Ohzawa, 2009; Hallum and Movshon, 2014; see also Ju et al., 2022). So, the difference in responses to first- and second-order visual stimuli can be attributed to two components of V1 neurons rather than to V1 and subsequent visual cortices.

Psychophysical studies suggest that the human visual system is sensitive to the second-order information (Baker and Mareschal, 2001; Chubb and Sperling, 1988; Chubb et al., 2001; Schofield et al., 2010) but note that the first- and second-order information are processed in separate mechanisms of the visual system (see also Cavanagh and Mather, 1989). Nishida et al. (1997) showed that the effect of motion adaptation only weakly transfers between the first- and second-order motion information (see also Nishida, 2011 for a review). The detection of the first- and second-order visual information is facilitated by additional visual information of the same type but not by additional information of a different type (Schofield and Georgeson, 1999). These results show that the first- and second-order information are processed differently in the visual system.

The calibration of the grayscale level of a display is necessary for this behavioral technique (see the subsection “2.3 Spatial frequencies and the magnocellular and parvocellular pathways”).

A grayscale image can be transformed to the first- and second-order visual stimuli (Figure 4). The stimuli are generated from a dynamic pattern of a 2D white noise by controlling the range of the white noise to represent the information in the grayscale image. The luminance of the noise pattern needs to be calibrated. For the first-order stimulus, the center of the noise range at each position in the pattern is proportional to the grayscale value at the same position in the image while the width of the range is constant across the pattern. The information in the grayscale image is represented by the 2D distribution of local mean luminance in the pattern. For the second-order visual stimulus, the width of the noise range is proportional to the grayscale value either positively or negatively while the center of the range is constant. The information in the grayscale image is represented by the 2D distribution of the local variance of luminance in the pattern. These transformations of the grayscale image can be implemented as image filters for an AR app. The grayscale image can serve as a control.

**FIGURE 4:**
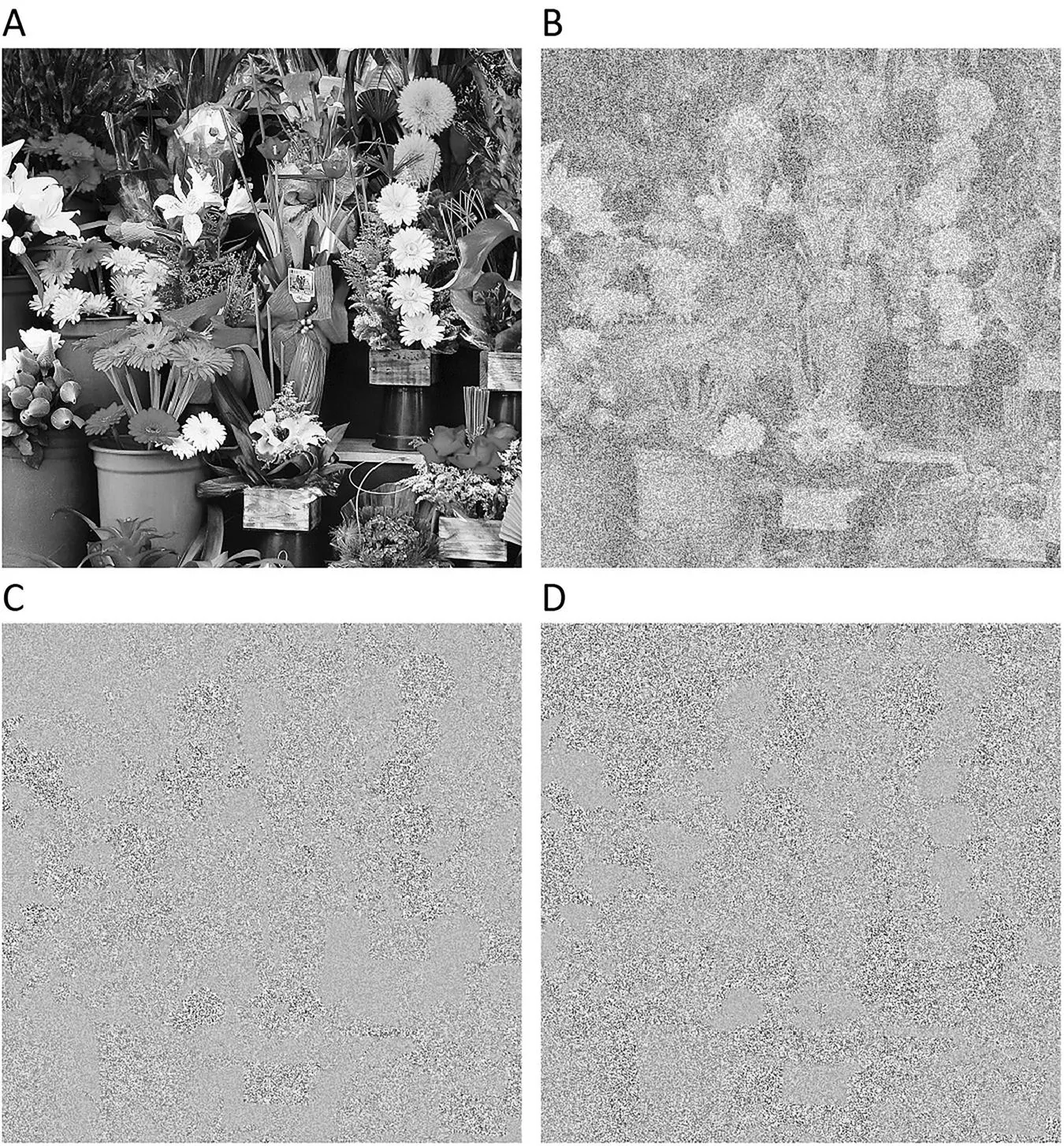
**(A)** A grayscale image of a real scene and **(B)** first-order and **(C,D)** second-order noise patterns that were generated from the grayscale image **(A)**. **(B)** The noise in the pattern is white noise and the center of its range is proportional to the grayscale value. **(C,D)** The width of the white noise range is **(C)** proportional and **(D)** negatively proportional to the grayscale value.

### 2.5 The power-law and the efficient coding in V1

Photo images of real scenes almost always have a common property in their spatial Fourier spectrums. The logarithm of their spatial-frequency spectrum is correlated linearly to the logarithm of the spatial frequency with a coefficient of around −1.2 (Figure 5; Field, 1987; Tolhurst et al., 1992). This property is referred to as a power law.

**FIGURE 5:**
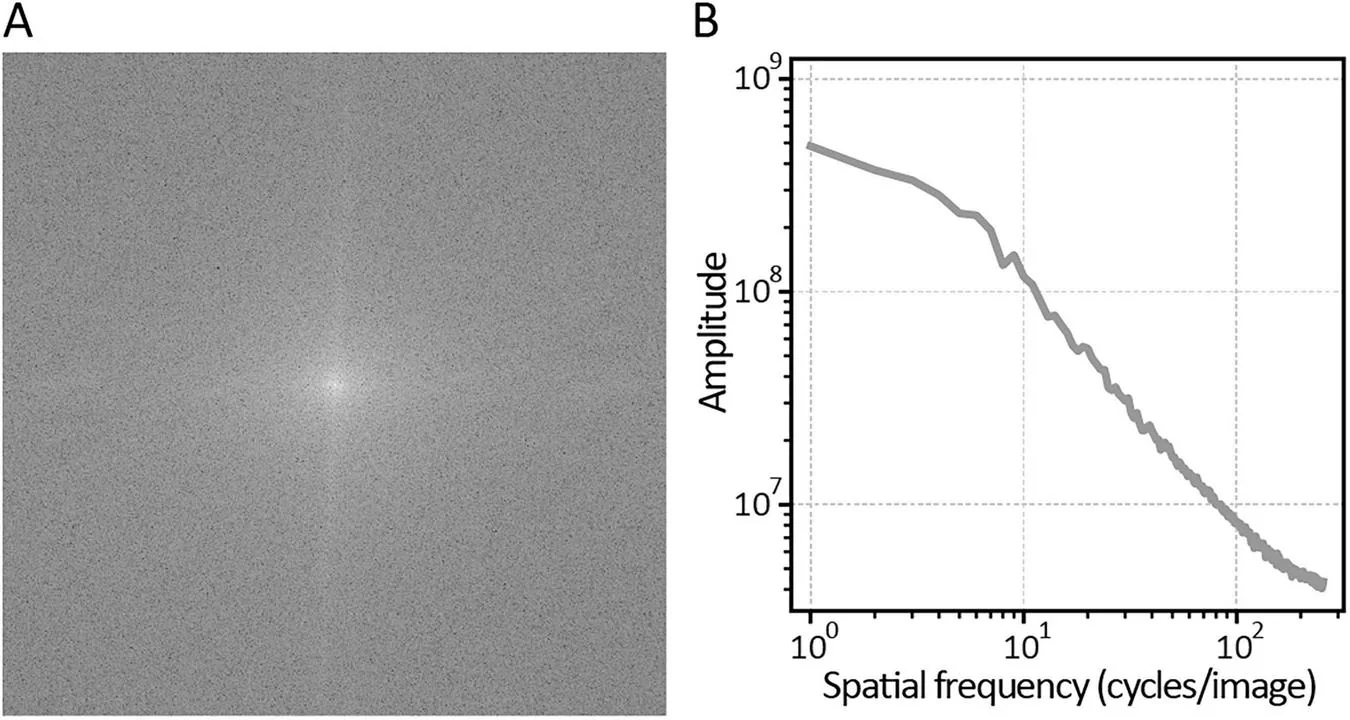
**(A)** The 2D Fourier transform (amplitude spectrum) of the image in Figure 4A and **(B)** the 1D amplitude spectrum that was generated by averaging the 2D distribution **(A)** across all orientations. **(A)** In the 2D pattern of the Fourier transform, a Fourier component is represented as a point in the pattern. The pattern is organized and a polar coordinate system is set with its origin at the center of the pattern so that the orientation and the spatial frequency of the component are represented by the radius and orientation of the position of the point representing the component in the pattern. The grayscale level at the point represents the log amplitude of the component.

The power law with the coefficient of −1.2 is important for the visual system. Neurophysiological studies in monkeys and mice suggest that V1 can process a retinal image more “efficiently” when the image satisfies the power law than when the image violates the law (Simoncelli and Olshausen, 2001; Baudot et al., 2013; Froudarakis et al., 2014; Rikhye and Sur, 2015; Yoshida and Ohki, 2020). A smaller number of neurons in V1 respond to the image satisfying the power law, spike rates of the individual neurons are smaller, and the timing of the spikes is more consistent across trials in which the same image is shown (Baudot et al., 2013; Rikhye and Sur, 2015; Yoshida and Ohki, 2020). Namely, the visual system can process the retinal image satisfying the power law with a small number of signals and a retinal image frequently satisfy the power law in a real environment. This allows the visual system to process more information with its finite resources (Attneave, 1954; Barlow, 1961; Simoncelli and Olshausen, 2001; see also Shannon, 1949). This efficient coding may not be intuitive because neuroimaging studies often interpret that a region of the brain is tuned to a property of stimuli when the region is activated more to stimuli with the property than to stimuli without the property.

The calibration of the grayscale level of a display is necessary for this behavioral technique (see the subsection “2.2 Spatial frequencies and the magnocellular and parvocellular pathways”).

Consider a grayscale image is given. To control whether the image violates (or satisfies) the power law, the Fourier transform of the image is computed and the magnitude of Fourier components at each frequency is scaled so that the power law is violated (or satisfied). Once this is done, the inverse-Fourier transform is computed to generate a transformed image that violates (or satisfies) the power law (Figure 6). The transformed image should be shown on a calibrated display. This transformation can be implemented as an image filter for an AR app but it is more computationally expensive than the filters previously discussed in this study because the Fourier and inverse-Fourier transforms are involved. It can be difficult to apply the transformation to an image of a real scene from a camera in real time or near-real time unless the AR app is running on a good processor.

**FIGURE 6:**
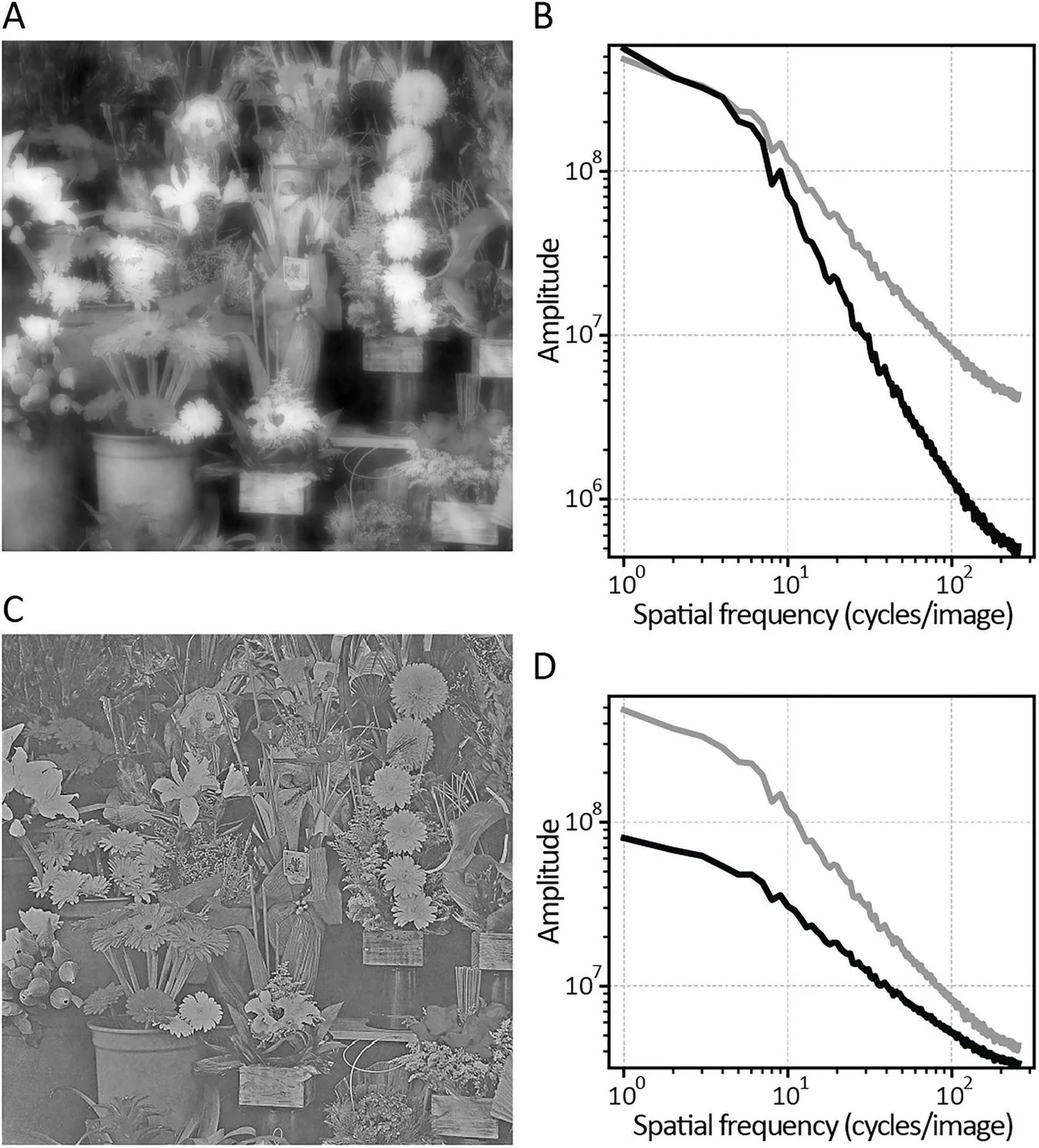
**(A,C)** Images that were generated by modifying the 1D amplitude spectrum (Figure 5B) of the image in Figure 4A so that the modified spectrums have **(B)** a steeper slope and **(D)** a shallower slope. The amplitude spectrum was raised to the power of panel **(B)** 1.5 for the image **(A)** and to the power of panel **(D)** 1/1.5 for the image **(C)**. The vertical positions of the spectra were adjusted so that the resulting images **(A,C)** have grayscale values between 0 and 255.

It is possible to transform an image of a real scene so that the transformed image violates or satisfies the power law by superimposing a noise pattern (Rikhye and Sur, 2015). Note that the original image of the real scene is expected to satisfy the power law. When the noise pattern violates the power law (e.g., white noise, Figure 7A), the image with the noise pattern also violates the law and the degree of the violation depends on the intensity of the noise pattern. When the noise pattern satisfies the power law (pink noise with its exponent is around 1.2, Figure 7B), the image with the noise pattern also satisfies the law and it does not depend on the intensity of the noise pattern. This transformation can be also implemented as an image filter for an AR app and its computational cost is low if the noise patterns are pre-rendered.

**FIGURE 7:**
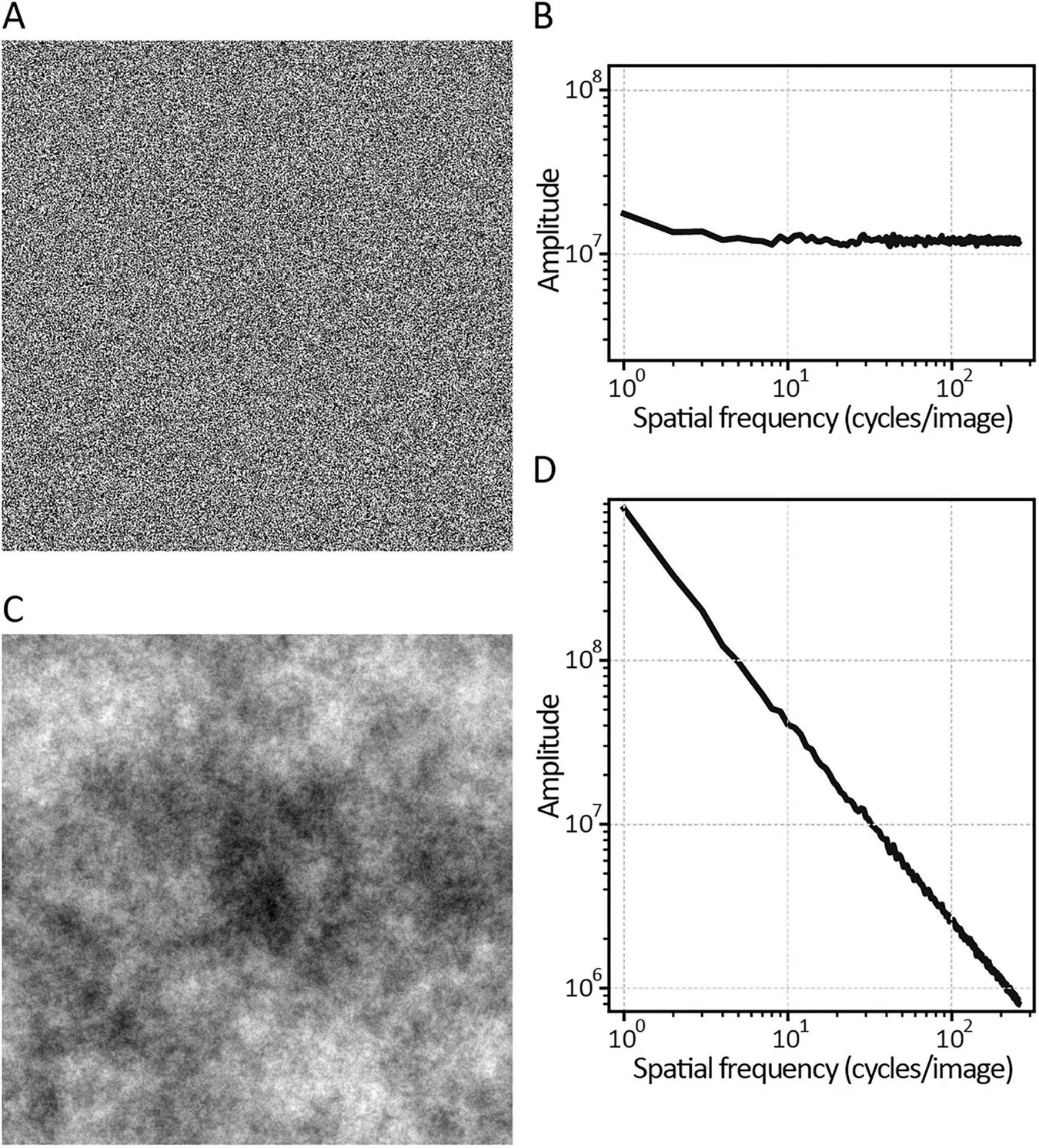
**(A)** A white noise image, **(B)** its 1D amplitude spectrum, **(C)** a pink noise image, and **(D)** its 1D amplitude spectrum. The amplitude spectrum **(B)** of the white noise image **(A)** is close to be flat. The pink noise image **(C)** was generated from the white noise image **(A)** so that the 1D amplitude spectrum **(D)** has a slope of approximately –1.2. Each amplitude of the spectrum of the white noise image was divided by a factor of its spatial frequency raised to the power of 1.2 to generate the spectrum of the pink noise image. The vertical position of the resulting spectrum **(D)** was adjusted so that the resulting image **(C)** has grayscale values between 0 and 255.

The grayscale image can serve as a control of this behavioral technique.

### 2.6 Regions in the retinae

The projection from the retina to V1 is systematically characterized by a 2D-to-2D log-polar transformation (Tootell et al., 1982). Systematic relationships with the retinae are also observed in V2, V3, and V4 (see Wandell and Winawer, 2011, Winawer and Witthoft, 2015 for reviews). Namely, specific regions of the early visual cortex can be stimulated by stimulating their corresponding regions of the retinae. The regions in the retinae also influence the subsequent processing of visual information in the brain.

The left and right cerebral hemispheres process various types of cognitive and perceptual information differently (see Ivry and Robertson, 1998; Toga and Thompson, 2003; Hervé et al., 2013 for reviews). The right and left sides of the retinae correspond to the left and right visual fields, respectively. The initial processing of visual information from the right and left sides of the retinae takes place in the left and right cerebral hemispheres, respectively (while the visual information from both halves of the retinal images are processed in both of the cerebral hemispheres after the initial processing). Then, the difference between the cerebral hemispheres can be studied by controlling visual stimuli in the right and left sides of the retinae individually (see the divided visual field paradigm, Bourne, 2006).

One side of the retina can be stimulated without stimulating the other side by using contact lenses with opaque regions that act as artificial scotomas. This technique was used to stimulate either the left or right side of the retina while occluding the other side from visual information (David et al., 1991; Dimond et al., 1975; Fouty et al., 1992; Sivak et al., 1985). One limitation of this method is that the occluded region cannot be changed dynamically. However, this issue may be resolved in the future with the development of smart contact lenses (Vásquez Quintero et al., 2020).

The right and left sides of the retina can be stimulated individually by using the HMD if the HMD is installed with an eye tracker and an image on the HMD’s screen can be rendered in real time or near-real time depending on an observer’s eye movement. Oculomotor information from the eye tracker is necessary because the regions of the screens of the HMD that correspond to specific regions of the retinae change depending on the orientation of the left and right eyes of an observer. There are HMDs with eye trackers but it is still difficult to render images on their screens in real time depending on the eye movement with sufficiently short delay and with sufficient precision for studying the visual system.

Note that the method of using contact lenses has also been used to study the visual processing of the peripheral region of the retina by occluding the central region of the retina (e.g., Rozhkova et al., 2019; Belokopytov et al., 2022). The central and peripheral regions of the retinae have different physiological properties, they are processed differently in the brain, and these differences are also reflected in the perception (Rozhkova et al., 2019; Belokopytov et al., 2022; Freeman and Simoncelli, 2011; Hadjikhani and Tootell, 2000; Ziemba and Simoncelli, 2021; see also Barton and Brewer, 2015; Wallis et al., 2019).

## 3 AR implementation of some of the behavioral techniques

Some of the behavioral techniques that can systematically modulate visual processing in the brain were implemented as image filters of an AR app (Figure 8). The image filters transformed a grayscale image (Figure 8B) into (i) a red-green isoluminant images (Figures 8C,D, see the subsection “2.1 Isoluminant stimuli and the dorsal pathway”) and (ii) first- and (iii) second-order noise images [Figures 8G–I, see the subsection “2.4 The first-order and second-order noise stimuli and the primary visual cortex (V1)”]. As controls, image filters that generated (iv) a monochrome-red image (Figure 8E) and (v) a monochrome-green image (Figure 8F) were also implemented. The app can also show (vi) the grayscale image (Figure 8B) and (vii) the full color image (Figure 8A) from which the grayscale image was generated. These monochrome-red and monochrome-green filters generate monochrome images by removing the green channel from the red-green isoluminant image and the red channel from the green-red isoluminant image.

**FIGURE 8:**
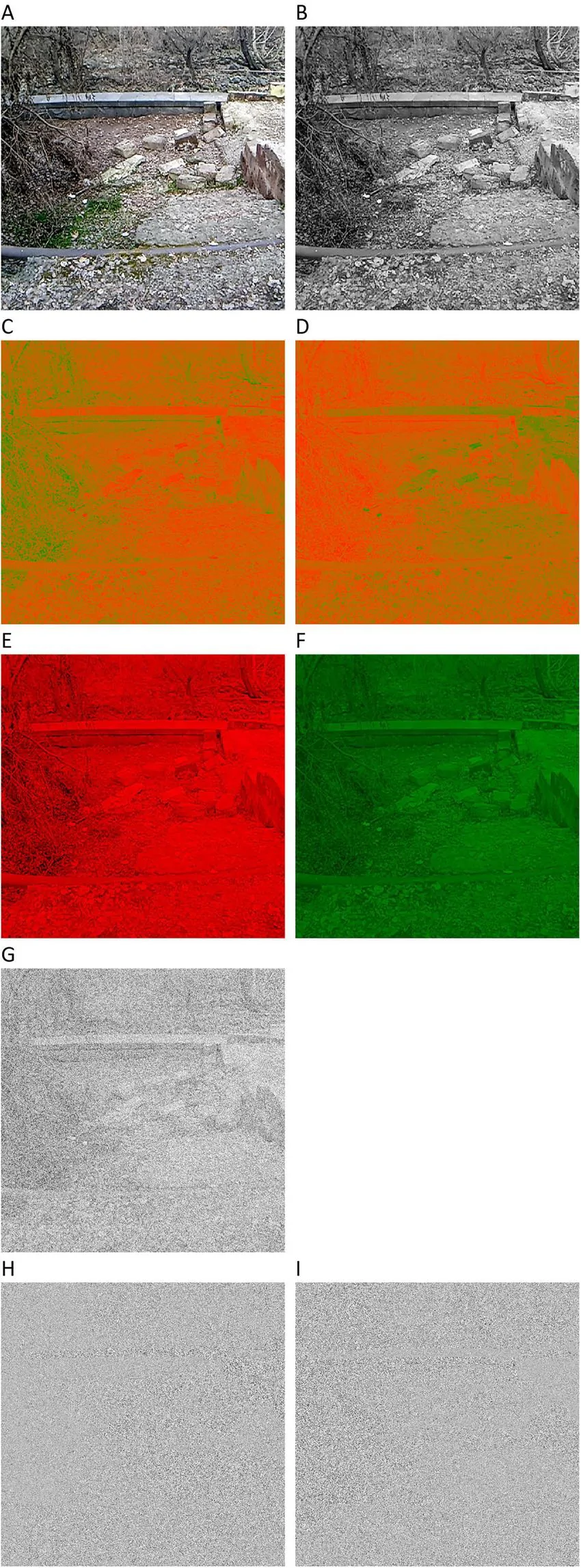
**(A)** The original full color photo and **(B)** its grayscale image of a real scene and **(C–I)** images that are generated from the grayscale image by applying image filters of Behavioral techniques implemented for AR HMDs. **(C,D)** Red-green isoluminant images. The relationships between the red-green scale and the grayscale in the two isoluminant images **(C,D)** are opposite from one another. **(E,F)** Red-black scale and green-black scale images that were generated by removing the green channel from the isoluminant image **(C)** and the red channel from the isoluminant image **(D)**. **(G)** A first-order noise pattern. The noise in the pattern is white noise and the center of its range is proportional to the grayscale value. **(H,I)** Second-order noise patterns. The width of the white noise range is **(C)** proportional and **(D)** negatively proportional to the grayscale value.

The app was installed in two AR HMDs composed primarily of a smartphone (Redmi 9A, 2020 and Lenovo Phab 2 Pro, 2016), a small wide-angle lens, and a wearable stereoscope (Figure 9). A camera of the smartphone located on the back of the screen was used to capture an image of a real scene in front of an observer wearing the AR HMD. The wide-angle lens was attached to the camera to widen the camera’s field of view (see Farshchi et al., 2021 for details). The image filter was applied to the captured image. Note that the screen of the smartphone was divided into two halves that were seen individually by the two eyes of an observer through the lenses of the stereoscope. These halves of the screen show two segments of the filtered image. The whole process of capturing the image of the real scene, applying the image filter to the captured image, and displaying the segments of the filtered image on the screen was carried out using the smartphone alone.

**FIGURE 9:**
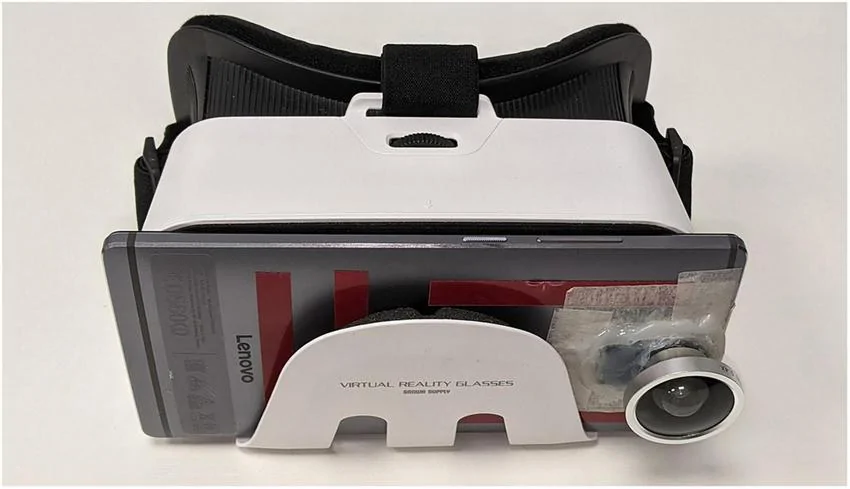
One of the AR head-mounted displays (AR HMD) used in this study.

The luminance of one of the AR HMDs was calibrated using the behavioral method (“see the subsection 2.1 Isoluminant stimuli and the dorsal pathway”). The gamma curves of the red and green channels were individually estimated and the ratio of luminance between the channels was estimated using a minimum motion technique (Anstis and Cavanagh, 1983). The gamma curve of the grayscale was also estimated. The calibration of this AR HMD was conducted using the behavioral method by one of the authors (MD). The luminance calibration of the other AR HMD was conducted using a luminance photometer.

The positions of the segments in the filtered image were adjusted so that the observer could easily fuse their retinal mages. Binocular depth cues (binocular disparity and vergence) from the retinal images of the image segments represented a frontoparallel plane with this set up but this depth information seemed to little deteriorate the immersive experience with the AR HMD (Wijntjes et al., 2016).

Although no stimulation was applied using the AR app, the set-up is compatible with pre-registered protocols in which brief offline TMS or short tES blocks precede an experimental session. This enables clean pre/post comparisons while keeping the wearable load minimal and within routine safety guidance (Huang et al., 2005; Woods et al., 2016).

The code of the AR app and its .apk file for a Google Android phone can be found at: https://osf.io/hz7b9/.

### 3.1 Observation

The AR HMDs with the image filters of the two behavioral techniques were tested by five of the authors (TS, AZ, and AT in Yerevan and MD and VK in Moscow).

The refresh rate of the AR HMDs was around 10 Hz, with a delay of around 300 ms. The refresh-rate (∼10 Hz) and the delay (∼300 ms) should be acceptable for an immersive experience when the AR HMDs were used, but it could degrade and limit an observer’s action and interaction (see Chen and Thropp, 2007; Cummings and Bailenson, 2016; Thropp and Chen, 2006 for reviews). This temporal property of the AR HMD was acceptable when observers slowly navigate in real scenes. Note that the smartphones used in this study were relatively old (2016, 2020) and more recent smartphones can process the filters faster.

It was difficult to perceive spatial information of a scene out there and to recognize objects from the isoluminant images much more than from the monochrome-red and -green images. The isoluminant gradient was especially ineffective for perceiving a 3D shape of an object and recognize it, like recognizing faces of people. This observation is consistent with the results of psychophysical experiments testing depth perception from isoluminant shading (Kingdom, 2003; Sunaga et al., 1995; see also Cavanagh and Leclerc, 1989; Kunsberg et al., 2018 for relevant studies).

The spatial perception with the first-order image and the recognition of objects were mostly possible but it was difficult to see details of their visual information because of the random dynamic noise added to the filtered image. They were almost impossible with the second-order image. Regions of low luminance contrast in the second-order image could be perceived as semi-transparent figures over a noisy background with high luminance contrast but this figure-ground segmentation of the second-order image was inconsistent with the veridical figure-ground segmentation. This effect was not expected.

A more reasonable comparison between the first- and second-order images may be possible if these images are generated from the depth distribution of the scene rather than the luminance distribution of an image of the scene. Obtaining the depth distribution of a real scene is challenging while this is relatively easy when the scene is virtual (i.e., VR instead of AR).

Photos of fields taken using the AR app were also uploaded to https://osf.io/hz7b9/.

## 4 General discussion

This study reviewed behavioral techniques that can systematically modulate visual processing in certain regions of the brain. These techniques are indirect but more affordable than more-direct techniques for stimulating the brain. The limitations of these techniques and the calibrations necessary for them were also discussed individually. Some of the behavioral techniques were implemented as image filters for AR HMDs as a proof-of-concept.

This study does not discuss the behavioral techniques as alternatives to more-direct techniques using neurophysiological devices. A limitation of the behavioral techniques is that regions of the brain that are modulated by these techniques are less selective and that their effects on the regions are not so systematic compared with the more-direct techniques. The effects of the behavioral techniques can also be affected by other properties of the visual information, as well as the observer’s tasks. Instead, the behavioral techniques are affordable and can reach people who cannot access the neurophysiological devices. The behavioral techniques can also be incorporated with the devices for a new research paradigm (Kveraga et al., 2007; Thomas et al., 2012).

The AR HMDs in this study were based on old smartphones (2016, 2020) and their temporal properties were a little sluggish: the refresh rate (∼10 Hz) of the smartphone’s display and the delay (∼300 ms) between changes in the real scene and their reflection on the display. This temporal property of the AR HMD was acceptable when observers interacted in real scenes based on static visual information, such as slowly navigating and some interactions with objects (see Sawada et al., 2022; Farshchi et al., 2021 for examples using a similar AR app). This temporal limitation is critical and it should be resolved to study human behavior based on dynamic visual information, such as natural movement, dynamic-object interaction, and visuomotor behavior in a real environment. It is difficult to study them without the AR HMD but it needs a sufficiently high refresh rate and short delay. The temporal property can be improved using a more recent smartphone and optimizing the code of the AR app. A real AR HMD with a fast processor can be also considered. They are more expensive than the smartphone-based HMD used in this study but they are still more affordable than almost any neurophysiological devices.

The study discussed AR but the image filters used for the behavioral techniques modulating visual processing in the brain can be also used in a Virtual Reality (VR). In a VR, a 2D image that is shown on the screen of an HMD is generated by rendering a virtual scene. The image filters can be applied to the image on the screen after an image is rendered and before it is displayed on the screen (“post-processing” in computer graphics). Note that VR requires data of the virtual 3D scene and the rendering process from the 3D scene to its 2D image while AR uses an image of a real scene captured by a camera. This allows us to deform the 3D scene and to distort geometry in the rendering process in VR to study the visual and cognitive systems (e.g., Chan et al., 2023; Szirmay-Kalos and Magdics, 2022; Hart et al., 2017; Pisani et al., 2019). AR and VR can be used depending on the purpose and control of the experiment.

A strength and potential risk factor of an AR/VR HMD is that it covers a large visual field of an observer. Especially when an image filter of a behavioral technique adds a temporal modulation of a retinal image, the possibility that it triggers a seizure in the observer with photosensitive epilepsy should be considered (Walter et al., 1946; Fisher et al., 2005; Harding et al., 2005; Trenité et al., 2025). This risk is particularly relevant for a high-contrast luminance modulation in a large field of view at a temporal frequency within a range of triggering photosensitive epilepsy. The image filter should be designed to minimize this risk (see International Telecommunication Union, 2023) for information on image properties that can trigger the epilepsy seizure). For example, consider adding a dynamic random noise pattern of luminance. Each element of the noise pattern should be sufficiently small that the average luminance within a reasonably large segment of the visual field remains nearly constant across frames, thereby minimizing large-area luminance modulation. Also, the range of the luminance change of the element should be minimized. Depending on the filters and visual stimuli used, researchers should consider screening individuals with a history of epileptic seizures during the recruitment phase and/or monitoring participants during experimental sessions. Other potential adverse effects of the use of AR/VR HMDs in experiments, such as cybersickness and physical injury, should also be considered (Madary and Metzinger, 2016; Kennedy et al., 2000; Zone, 2012).

A pragmatic next step is a hybrid protocol of AR/VR and non-invasive brain stimulation. Short, portable tES sessions can be paired with specific image filters of AR/VR to probe hypothesized pathways, for example testing whether occipito-parietal stimulation modulates performance costs under isoluminance, or whether motor-area tACS (Pozdniakov et al., 2021; Feurra et al., 2019) influences visually guided locomotion. This design maintains causal leverage while preserving ecological validity and low cost (Charvet et al., 2015; Fertonani and Miniussi, 2017; Polanía et al., 2018).

An AR/VR HMD with the image filters modulating visual processing in certain brain regions allows us to study the roles of these regions in visual perception under dynamic conditions and in the interaction between perceptual and motor processes on human behavior, such as motor control with visual feedback. When an observer sees a 3D scene with moving the observer’s head, the retinal image of the scene changes. The visual system perceives depth from the relationship between the head movements and the image changes (motion parallax). The image changes can also be important for the perception of materials (Anstis, 1992; Blake and Bülthoff, 1990). Depth perception from motion parallax has been studied with restricting the directions and ranges of the head movements because of the technical limitation of head tracking as well as the control of their experiments. This technical limitation can be resolved by using the AR/VR HMD. With the HMD, it is also possible to test the effect of the dynamic head movements on more complex cognitive tasks, such as face recognition and finding a familiar face in a crowd. An interesting example is visual search and foraging. Gilchrist et al. (2001) reported that an observer uses different strategies for visual search and foraging where the observer can freely move around. The AR/VR HMD may be also useful in more industry-oriented applications, like the evaluation of outdoor advertisement and the urban environment.

There can be potential clinical usage of the behavioral techniques modulating visual processing. An AR HMD with the image filter of S-cone isolating stimuli can be useful in studying the roles of the superior colliculus in people’s social behavior. The superior colliculus processes an emotional signal in visual information (Carretié et al., 2021; Méndez et al., 2022), which is important for the social behavior, including interaction with others. Note that some of experiments in social psychology that involve this interaction are conducted in real environments. The wearable AR HMD can be useful to study how the brain regions affect the social behavior in these experiments (most AR HMDs are bulky so they are likely limited to use in interactions with experimenters or confederates at the current stage). The superior colliculus is especially sensitive to negatively-affective visual information (Almeida et al., 2015; Wang et al., 2020; Ajina et al., 2020). Then, the filter of the S-cone isolating stimuli may be used to control the intensity of exposure therapy for post-traumatic stress disorder (PTSD) and phobias. Some other clinical issues are associated with brain regions whose activity can be modulated using the behavioral techniques discussed in this study: e.g., the superior colliculus with autism (Jure, 2019, 2022), the magnocellular- or dorsal-pathways with dyslexia (Quercia et al., 2013; Gori and Facoetti, 2015) and schizophrenia (Whitford et al., 2018; Kody and Diwadkar, 2022), and the parvocellular pathway with amblyopia (Wen et al., 2021; see Gao et al., 2025; Zhou et al., 2025 for examples). Note that the clinical usage of the behavioral techniques discussed in this paragraph remains speculative and requires careful clinical validation and evaluation of the safety of the techniques and the HMD, especially for vulnerable groups.^2^

The behavioral techniques that were discussed in this study only indirectly modulate visual processing in certain regions in the brain, and their relationship with the modulated regions is based on the results of neurophysiological studies. So, the relationship may be revised in the future, which could affect the neurophysiological inferences drawn from the results of the experiments that used the techniques. Besides, it has been shown that the techniques clearly affect visual perception, which is the outcome of the process in the brain. Therefore, the functional properties of the process can still be discussed in terms of which aspects of perception are or are not affected by the techniques.

## Footnotes

1 It is worth pointing out that the relationship between a physical luminance measurement and a radiant energy distribution is also based on psychophysical data of past studies (see Bertenshaw, 2020; Sharpe et al., 2005 for reviews).

2 Any translation into clinical practice should be carried out under appropriate ethical oversight, with informed consent, clinical supervision, and safety monitoring, especially when vulnerable populations are involved. Additional safeguards should be considered for applications in low-resource or field settings, such as adequate clinical supervision, referral pathways, privacy protections, and culturally appropriate implementation.
